# Construction of Pt@BiFeO_3_ Xerogel-Supported O-g-C_3_N_4_ Heterojunction System for Enhanced Visible-Light Activity towards Photocatalytic Degradation of Rhodamine B

**DOI:** 10.3390/gels9060471

**Published:** 2023-06-08

**Authors:** Abubakar Usman Katsina, Sonia Mihai, Dănuţa Matei, Diana-Luciana Cursaru, Raluca Şomoghi, Cristina Lavinia Nistor

**Affiliations:** 1 Faculty of Petroleum Technology and Petrochemistry, Petroleum—Gas University of Ploiești, 100680 Ploiești, Romania; 2 Department of Pure and Industrial Chemistry, Bayero University, Kano PMB 3011, Nigeria; 3 National Institute for Research and Development in Chemistry and Petrochemistry—ICECHIM, 060021 Bucharest, Romania

**Keywords:** xerogels, rhodamine B, S-scheme heterojunction photocatalyst, bismuth ferrite (BiFeO_3_), graphitic carbon nitride (g-C_3_N_4_), perovskites, hydrothermal method, photodegradation

## Abstract

Synthetic organic pigments from the direct discharge of textile effluents are considered as colossal global concern and attract the attention of scholars. The efficient construction of heterojunction systems involving precious metal co-catalysis is an effective strategy for obtaining highly efficient photocatalytic materials. Herein, we report the construction of a Pt-doped BiFeO_3_/O-g-C_3_N_4_ (Pt@BFO/O-CN) S-scheme heterojunction system for photocatalytic degradation of aqueous rhodamine B (RhB) under visible-light irradiation. The photocatalytic performances of Pt@BFO/O-CN and BFO/O-CN composites and pristine BiFeO_3_ and O-g-C_3_N_4_ were compared, and the photocatalytic process of the Pt@BFO/O-CN system was optimized. The results exhibit that the S-scheme Pt@BFO/O-CN heterojunction has superior photocatalytic performance compared to its fellow catalysts, which is due to the asymmetric nature of the as-constructed heterojunction. The as-constructed Pt@BFO/O-CN heterojunction reveals high performance in photocatalytic degradation of RhB with a degradation efficiency of 100% achieved after 50 min of visible-light irradiation. The photodegradation fitted well with pseudo-first-order kinetics proceeding with a rate constant of 4.63 × 10^−2^ min^−1^. The radical trapping test reveals that h^+^ and ^•^O_2_^−^ take the leading role in the reaction, while the stability test reveals a 98% efficiency after the fourth cycle. As established from various interpretations, the considerably enhanced photocatalytic performance of the heterojunction system can be attributed to the promoted charge carrier separation and transfer of photoexcited carriers, as well as the strong photo-redox ability established. Hence, the S-scheme Pt@BFO/O-CN heterojunction is a good candidate in the treatment of industrial wastewater for the mineralization of organic micropollutants, which pose a grievous threat to the environment.

## 1. Introduction

Contamination of water by lethal organic micropollutants (OMPs) represents a critical problem that causes a global threat to susceptible aquatic ecosystems, human health, and sustainable economic development [1,2,3,4]. These OMPs consist of a large collection of recalcitrant and ecotoxic species of anthropogenic origins that are biologically active in aquatic environments at trace concentrations [5]. Typical OMPs whose exponential existence is attributed to the industrial revolution and rapid population growth include synthetic organic dyes, phenols, fertilizers, cosmetics, pharmaceuticals, pesticides, and estrogens [6,7,8,9]. The health risks of exposure associated with these intractable OMPs include genotoxicity, neurotoxicity, ecotoxicity, carcinogenicity, mutagenicity, immune response disorders, antibiotic resistance, and infertility [10,11,12,13]. Meanwhile, synthetic organic dyes (SODs) that are discharged from the textile industry are considered to be among the foremost water-polluting toxins globally [14]. Rhodamine B (RhB), a crucial textile dyestuff that can also be used to dye various substances such as dye lasers, paints, stamp pad inks, and carbon sheets is one of such hazardous dye effluents [15], and its increased accumulation in water can be associated to its highly persistent nature in the aquatic environment and non-biodegradability [16]. The numerous toxic effects and damage to sustainable economic growth associated with the dye make its removal from water indispensable.

Conventional wastewater decontamination technologies such as membrane filtration, adsorption, electrodeionization, precipitation, and electrodialysis suffer huge setbacks that include incomplete mineralization of pollutants, harmful by-product formation, and consumption of large amounts of energy [17,18,19,20,21,22]. In contrast, heterogeneous photocatalytic degradation of water-polluting OMPs employing different semiconductors under visible-light irradiation is promising, cost-effective, and eco-friendly [23,24]. Photocatalytic degradations are initiated by photoexcited electron/hole (e−/h+) pairs with the formations of reactive free radicals that react with the surface-adsorbed contaminants, converting them to harmless products. Thus, the efficiency of photocatalysis depends fundamentally on the ability of the photocatalytic material to efficiently absorb light in a wide wavelength range and produce charge carriers that live long enough to form reactive radicals.

It is well established that constructing heterojunction photocatalysts is a reliable approach through which the charge separation of photogenerated carriers can be efficiently enhanced [25]. Moreover, effective charge separation of charge carriers boosts photodegradation efficiency due to improved quantum yields of photo-assisted catalytic reactions. The generation of the internal electric field is an important factor that enhances photocatalytic activity, and hypothetically, the incorporation of dissimilar semiconductors creates an in-built electric field at the interface formed between the integrated semiconductors. This inner electric field drives the electrons and holes from the conduction band of the higher-oxidation-potential semiconductor to the valence band of the higher-reduction-potential semiconductor, leaving the photoexcited holes in the former semiconductor for oxidation half-reaction and the photogenerated electrons in the latter for reduction half-reaction. Recently, Lan et al. reported a successful synthesis and enhanced photodegradation activity of g-C_3_N_4_/BiVO_4_/CNTs composites [26]. Similarly, Shi et al. accelerated the photocatalytic degradation and mineralization of oxalic acid through band gap modification of g-C_3_N_4_ with AgCl [27]. In another development, a remarkable promotion in solar-driven photocatalytic activity towards RhB degradation over Bi_2_WO_6_/g-C_3_N_4_ composites was reported by Chen et al. [28]. Thus, the choice of an appropriate semiconductor and the construction of effective heterojunctions with metal-free CN may perhaps be significant in the improvement of the inherent photocatalytic activity.

Bismuth ferrite (BiFeO_3_, BFO) is a multiferroic, magneto-electric mixed metal oxide semiconductor and the most widely reported photovoltaic ferroelectric perovskite material [29]. It has attracted great attention as a unique material category in heterogeneous visible-light-assisted catalysis due to its narrow band-gap energy (Eg; 2.2 eV), low toxicity, and excellent chemical stability [30,31]. Additionally, the performance of ferroelectric BFO photocatalysts in the degradation and mineralization of water contaminants is considered admirable when compared with other photo-responsive semiconducting materials [32]. However, besides other downsides of BFO such as particle agglomeration and variable perovskite phase during preparation, its photocatalytic activity is constrained owing to its sluggish charge transfer kinetics, poor reproducibility, and recombination rate of photogenerated charge carriers [33]. Several studies have reported that one of the effective ways of enhancing its photocatalytic efficiency is through the construction of BFO-based heterostructured composites with other semiconducting materials such as plasmonic metals (Pt/BFO, Au/BFO, Ag/BFO) and polymeric carbon nitride (BFO/g-C_3_N_4_). The co-loading of Pt can reduce the rate of recombination of charge carriers, which thus enhances the catalyst performance efficiency.

Recently, two-dimensional graphitic carbon nitride (g-C_3_N_4_, CN) has attracted wide research interest as a catalyst support candidate in the design and applications of visible-light photocatalytic materials [34]. It exhibits outstanding thermochemical stability due to its metal-free nature, while its suitable electronic band configuration and pi-conjugation improve the generation, mobility, and conductivity of charge carriers [35]. These exceptional physicoelectrochemical properties make it a compelling candidate for visible-light photocatalytic applications. Evidently, several studies reported performance investigation of BFO-supported CN heterojunction photocatalysis, with such systems demonstrating better photocatalytic performance in visible-light applications. For example, Wang et al. reported Pt-doped CN nanosheets with enhanced photocatalytic activity towards the water-splitting reaction [36]. However, the photocatalytic efficiency of pristine CN is restricted by its low surface area, fast recombination rate of photoexcited electron–hole pairs, narrow visible-light harvesting range, and charge separation efficiency. Therefore, the need to promote its efficiency through charge density modulation in the CN is inevitable. The use of oxidants with lower redox potentials such as H_2_O_2_ to treat CN could prevent its destruction and modify its characteristic surface, resulting in an enhancement in its photocatalytic activity [37]. Ilkaeva et al. reported a successfully treated CN surface with improved photocatalytic activity under solar light [38].

Co-catalyst loading is one of the effective strategies for enhancing the charge transfer rate [39,40,41]. Recently, co-catalysis with metals such as Pt, Au, and Ag with unique surface plasmon polaritons (SPPs) and the surface plasmon resonance (SPR) effect have been reported to improve the interaction of light and other materials [42,43]. In photocatalysis, the SPR effect has the benefits of intensifying the light response range, enhancing appropriate utilization efficiency of the light spectrum, and providing additional reaction sites through electron trapping that in turn elongate the lifespan of plasmonic charge pairs [44]. As co-catalysts, they form a Schottky junction with other semiconductor catalysts. They improve the generation and separation of charge carriers via the synergistic effect of SPR and the Schottky junction in the plasmonic metal–semiconductor heterojunction composites [45]. Pt is a typical example of precious metal, and it has been widely reported alongside Au and Ag for plasmonic catalysis due to its large work function and high activity compared to other metals in that category [46].

Herein, we report the construction of Pt@BiFeO_3_/O-g-C_3_N_4_ heterojunction system via an ultrasonic–hydrothermal synthesis route. The rationale of the work is to enhance the photocatalytic efficiency through synergistic property contributions of the participating semiconductors and the LSPR effect of plasmonic Pt metal. Meanwhile, the textural, structural, thermal, morphological, and optical properties were characterized using various physicochemical characterization techniques, whereas the photocatalytic performance of the as-constructed heterojunction was investigated against aqueous RhB degradation under visible-light irradiation. Various proportions of BiFeO_3_ xerogel and the O-g-C_3_N_4_ support (BiFeO_3_: O-g-C_3_N_4_; 25:75, 50:50, and 75:25) were prepared for optimization before the incorporation of the metallic Pt species. The highest photodegradation efficiency of RhB was recorded on the 0.5 wt.% Pt@BFO_50_/O-CN_50_ heterojunction system with a 1:1 proportion of xerogel and support. The effects of important parameters such as catalyst dosage, initial concentration of pollutant, and active species scavenging on RhB degradation under visible-light illumination were investigated.

## 2. Results and Discussion

### 2.1. X-ray Diffraction (XRD) Measurements

**Figure 1:**
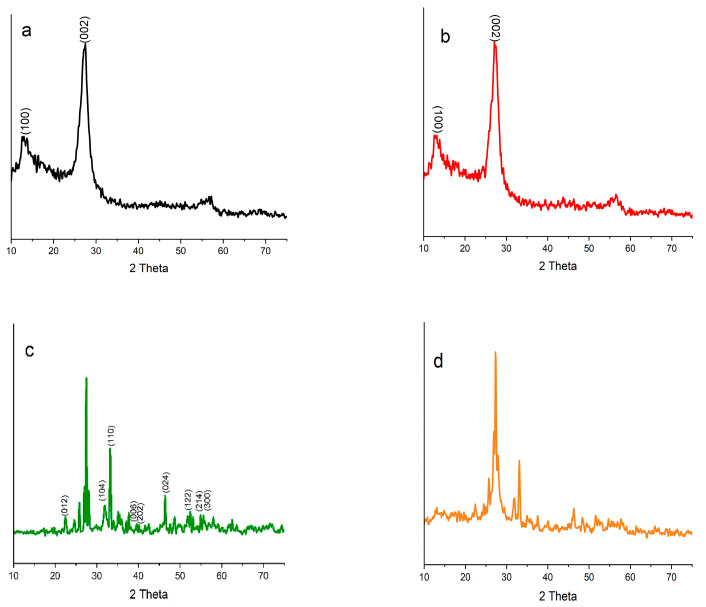

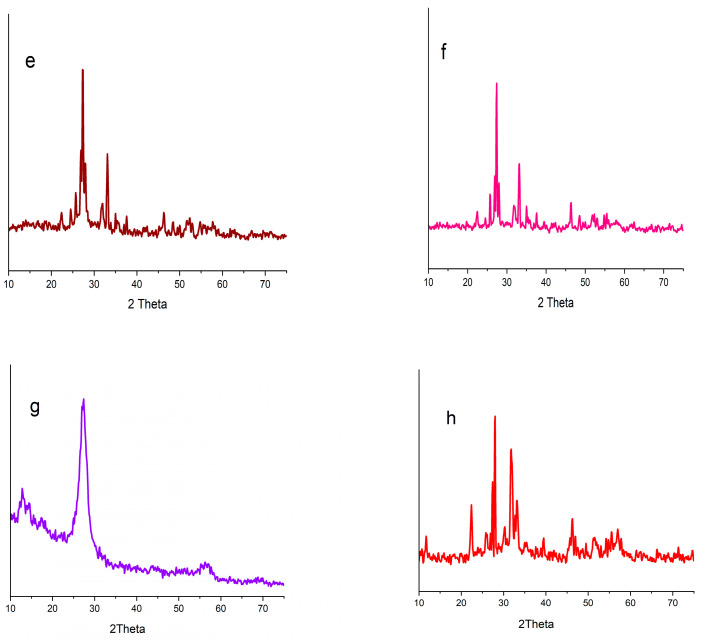
XRD diffraction patterns of (**a**) O-CN, (**b**) BFO, (**c**) BFO_25_/O-CN_75_, (**d**) BFO_50_/O-CN_50_, (**e**) BFO_75_/O-CN_25_, (**f**) Pt@BFO_50_/O-CN50, (**g**) Pt@O-CN, and (**h**) Pt@BFO samples.

Figure 1a–f present the room temperature XRD patterns of BFO, Pt@BFO, O-CN, Pt@O-CN, BFO_25_/O-CN_75_, BFO_50_/O-CN_50_, BFO_75_/O-CN_25_, and Pt@BFO_50_/O-CN_50_ samples in the range of 15 < 2θ < 75. The well-crystalline diffraction peaks in Figure 1b at 22.4°, 32.0°, and 46.5° corresponding to the (012), (110), and (004) planes can be indexed by the perovskite phase characteristic of BFO [47]. Trace amounts of Bi_2_Fe_9_O_4_ and Bi_25_FeO_40_ impurities were also seen due to the volatile nature of Bi during BFO fabrication [48]. For the O-CN sample, the broad diffraction peaks at 13.05 and 27.4° 2θ values in Figure 1a can be well indexed to the (100) and (002) diffraction planes of hexagonal g-C_3_N_4_ with an interplanar distance of 0.325 Å [49]. After BFO and O-CN incorporation, the diffraction peaks of both BFO and O-CN phases were noticeably observed, which firmly indicates the coupling of BFO and O-CN. As displayed in Figure 1c–e, the relative intensities of the (104) and (110) Bragg peaks decreased after the incorporation with O-CN. However, it can be observed from the XRD patterns in Figure 1f that incorporation of Pt toBFO_50_/O-CN_50_ did not appreciably change the reflections indexed to both BFO and graphitic planes, which implies that the incorporation of Pt has no influence on the phase structure of BFO and CN, or there is a limited amount of Pt in the as-constructed photocatalyst [50,51].

### 2.2. FT-IR Measurements

**Figure 2:**
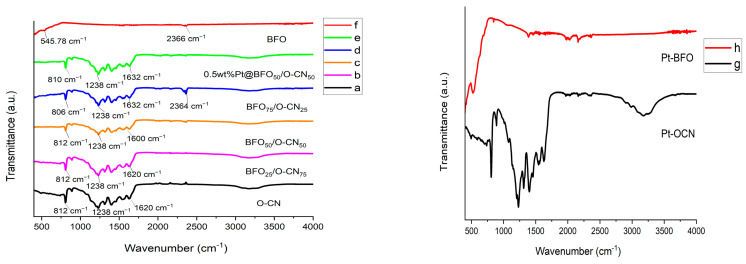
FT-IR spectra of the samples.

Figure 2 displays the FT-IR spectra of BFO, O-CN, various compositions of BFO/O-CN, and the Pt@BFO/O-CN photocatalyst. In the case of O-CN, the bands in the range of 1238–1620 cm^−1^ and 812 cm^−1^ as shown in Figure 2a were associated with the stretching vibrations of C-N and C=N and bending vibration of C-N heterocycles, respectively [52]. The broad band assigned in the range of 3050–3450 cm^−1^ confirmed the stretching vibration of the N-H bond due to the existence of amino groups [53]. For the spectrum of pristine BFO in Figure 2f, the noticeable absorption peaks positioned at around 445 and 536 cm^−1^ can be indexed to the Fe-O stretching and bending vibrations, respectively [54]. For the different BFO/O-CN composites in Figure 2b–d, the Pt-O-CN in Figure 2g, and the Pt-doped BFO/O-CN in Figure 2e, all the characteristic vibrational peaks of CN were visible, signifying that the interlaying stacking structure of O-CN is preserved, which corroborates the XRD results. Additionally, a small shift and decrease in intensity are observed in Figure 2b–e for the bands at 812 cm^−1^, which suggests a strong interaction between BFO and O-CN.

### 2.3. Morphology Studies

**Figure 3:**
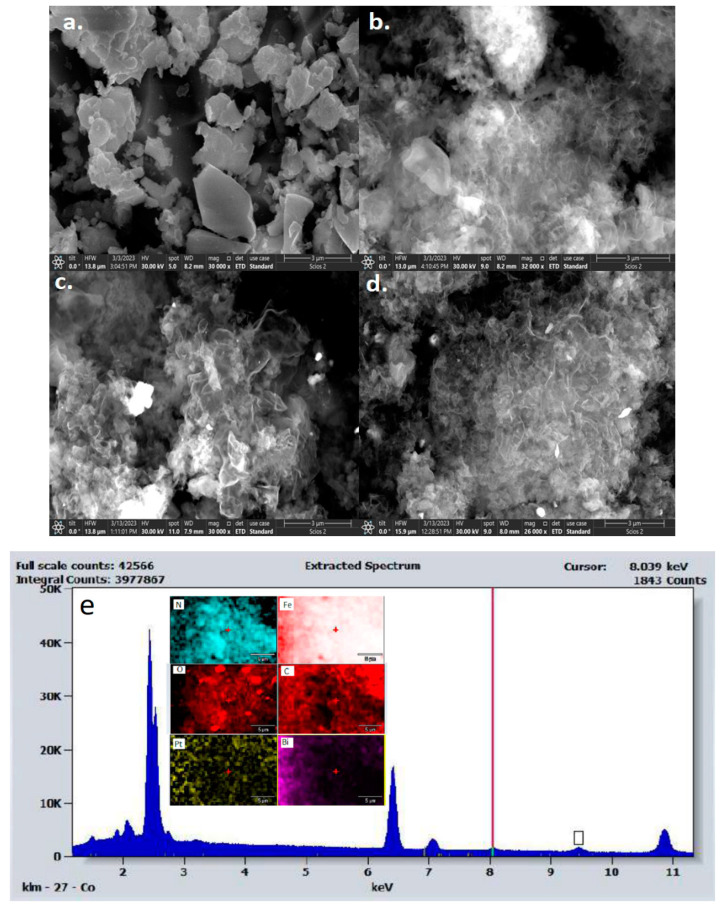
SEM micrographs of (**a**) BFO, (**b**) O-CN, (**c**) BFO_50_/O-CN_50_, and (**d**) Pt@BFO_50_/O-CN_50_; (**e**) EDS mapping of Pt@BFO_50_/O-CN_50_.

The SEM images of the as-synthesized BFO, O-CN, BFO_50_/O-CN_50_, and Pt@BFO_50_/O-CN_50_ samples are shown in Figure 3 below. Figure 3b shows that the O-CN exhibits a stacking flat structure with furrows of about 3.5 μm lateral size. The irregular, hollow layered structure of the g-C_3_N_4_ phase of O-CN dominates the morphologies of the BFO_50_/O-CN_50_ samples in Figure 3c and Pt@BFO_50_/O-CN_50_ samples in Figure 3d, signifying that the composite samples consisting of BFO and Pt are homogeneously distributed in the two-dimensional porous structure of O-CN. Additionally, the incorporation of Pt can be confirmed from the EDS mapping in Figure 3e below.

### 2.4. Sorption Studies

**Figure 4:**
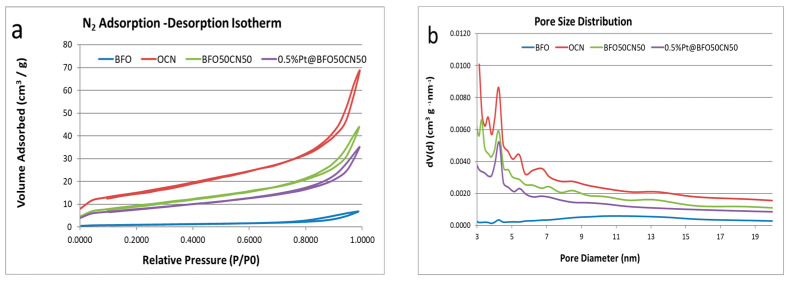
Textural analysis of the as-prepared samples: (**a**) N_2_ physisorption isotherms; (**b**) BJH pore size distribution.

Figure 4 displays N_2_ adsorption–desorption isotherms and pore size distributions for BFO, O-CN, BFO_50_/O-CN_50_, and 0.5 wt.% Pt@BFO_50_/O-CN_50_ samples obtained at 77 K. All the isotherms in Figure 4a demonstrate type IV isotherms with characteristic H3 hysteresis loops, which indicate the formation of a mesoporous structure of the as-fabricated materials [55]. Table 1 presents the surface area, pore volume, and average pore diameter of the as-synthesized materials. As shown in the table, the BET surface area of pristine O-CN is higher than that of BFO_50_/O-CN_50_ composites. On the other hand, the specific surface area of the Pt-doped BFO_50_/O-CN_50_ sample is lower than that of the BFO_50_/O-CN_50_ composites. Thus, the BET specific surface area of the samples increases in the following order: BFO <  Pt@BFO_50_/O-CN_50_  <  BFO_75_/O-CN_25_ <  BFO_50_/O-CN_50_ < BFO_25_/O-CN_75_ < O-CN. The lower surface area detected for the Pt-doped BFO_50_/O-CN_50_ compared to the undoped BFO_50_/O-CN_50_ composite might be due to Pt particles occupying the pore sites of the as-constructed photocatalytic system. However, the mesoporous structural ordering of the photocatalyst was not altered with the doping of the Pt species, which corroborates the results from XRD and FT-IR analyses.

### 2.5. Photocatalytic Degradation of RhB

**Figure 5:**
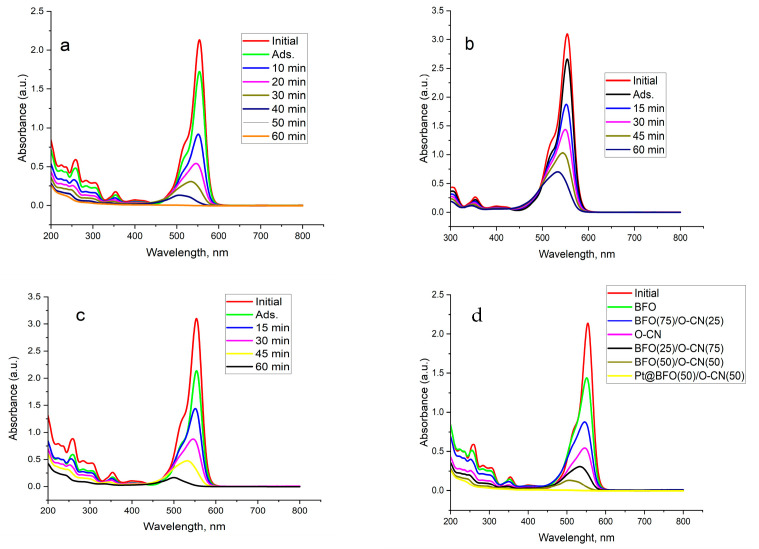

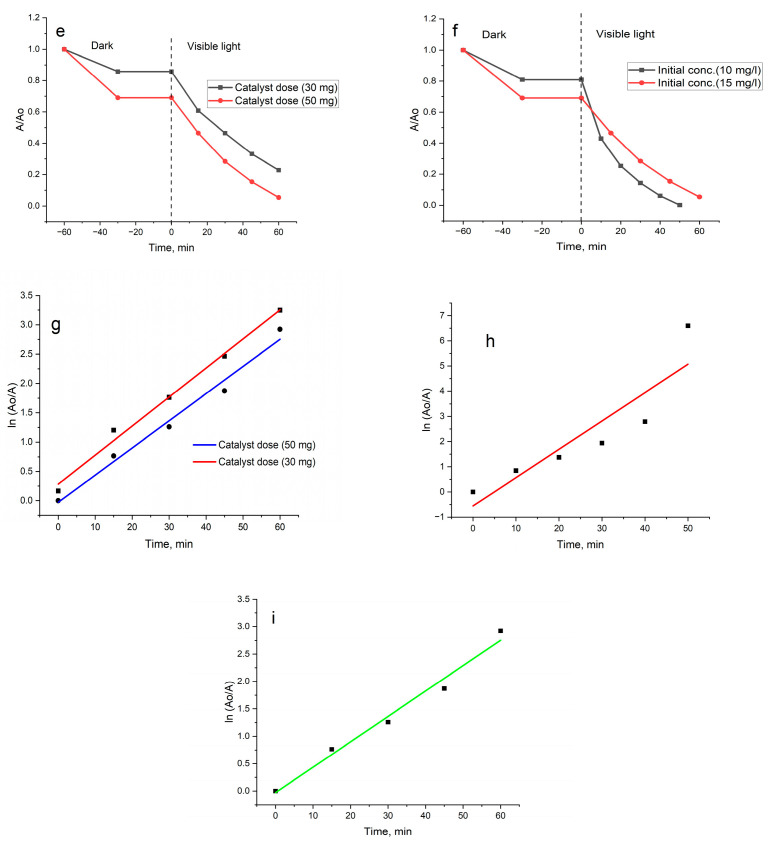
Absorbance data of RhB decomposition for 0.5 wt.% Pt@BFO50/O-CN50 at (**a**) 10 mg/L dye conc. and 50 mg catalyst dose, (**b**) 15 mg/L dye conc. and 30 mg catalyst dose, and (**c**) 15 mg/L dye conc. and 50 mg catalyst dose; (**d**) comparison of various catalysts; photocatalytic degradation of RhB in the presence of (**e**) 30 and 50 mg catalyst dose and (**f**) 10 mg/L initial conc. of RhB; pseudo-first-order kinetics of (**g**) 30 and 50 mg catalyst dose. (**h**) 10 mg/L initial conc. of RhB, and (**i**) 15 mg/L initial conc. of RhB.

Figure 5 below displays the results of the as-constructed 0.5 wt.% Pt@BFO_50_/O-CN_50_ heterojunction photocatalyst in photodegradation of aqueous RhB dye. The photodegradation of contaminant was first carried out in the dark to establish adsorption–desorption equilibrium. The results show that the photocatalyst adsorbs a portion of the dye owing to the surface pores of the as-constructed catalyst. Thereafter, the photodegradation proceeded in the presence of light. Operational parameters such as catalyst dose (30 and 50 mg), initial concentration of RhB (10 and 15 mg/L), and photodegradation time (0–50 min at 10 min intervals for 10 mg/L; 0–60 min at 15 min intervals for 15 mg/L) were investigated at the pH of 6.65.

The effects of catalyst doses of 30 and 50 mg on the RhB photodegradation were investigated, and the results are displayed in Figure 5b,c. A remarkable improvement in the mineralization of RhB (15 mg/L) was observed with the increase in the amount of the catalyst, with photocatalytic efficiency increasing from 77.2% for the catalyst dose of 30 mg in 60 min to about 94.6% for the 50 mg catalyst dose at the same time. Conversely, when the catalyst dose was further increased to 100 mg, a sharp decline was observed in RhB degradation, with efficiency declining to about 59.2% in 60 min. For this reason, the presence of the 0.5 wt.% Pt@BFO_50_/O-CN_50_ heterojunction catalyst under visible light could obviously improve the production of h^+^ and e^-^ at a low dose, enhancing the mineralization of RhB pollutant, whilst at a higher dose, there may be a restricted light infiltration into RhB solution due to self-aggregation of the catalyst. The RhB photodecomposition at 10 and 15 mg/L initial concentrations is presented in Figure 5a,c below. The photocatalytic efficiency of RhB degradation declined from about 100% in 50 min to about 94.6% in 60 min when the initial RhB concentration increased from 10 to 15 mg/L. However, a drastic decrease in efficiency was noted at 50 mg/L. This might be attributed to the saturation of accessible active sites of the Pt@BFO_50_/O-CN_50_ catalyst by RhB molecules at a higher initial concentration. Therefore, the initial RhB concentration of 10mg/L was chosen in this work to replicate practical conditions and to ensure that a sufficient amount of RhB decomposition is achieved in a short time.

For comparison, the performances of a pristine BFO xerogel, O-CN, BFO_25_/O-CN_25_, BFO_50_/O-CN_50_, and BFO_75_/O-CN_25_ were also investigated. The outcomes in Figure 5d elucidate that the BFO xerogel and O-CN can photodecompose RhB after 50 min of visible-light irradiation with corresponding photocatalytic efficiencies of 32.9% and 74.6%, whilst the RhB photodegradation for BFO_25_/O-CN_75_, BFO_50_/O-CN_50_, and BFO_75_/O-CN_25_ exhibited the photocatalytic efficiency of 85.6%, 94%, and 58.8%, respectively, after the same time of irradiation. An increase in the photodegradation efficiency of RhB was detected from 60% for BFO_25_/O-CN_75_ to 94% for BFO_50_/O-CN_50_, while a poor efficiency was recorded for BFO_75_/O-CN_25_. This might be related to the differences in surface area and pore volume. Therefore, the binary BFO_50_/O-CN_50_ catalyst was selected for the incorporation of the plasmonic Pt metal. The low efficiency in the BFO and O-CN can be attributed to the fast recombination of photogenerated charge carriers within the pristine photocatalysts. In a closer examination of the absorbance peak, a blueshift of the maximum intensity from 550 to 543 nm was observed, pointing to intermediate species formation upon either removal of ethyl groups or cleavage of the conjugated chromophore [56].

#### 2.5.1. Kinetics

Figure 5 shows the kinetics plot for the photodegradation of aqueous RhB in the presence of 0.5 wt.% Pt@BFO_50_/O-CN_50_ heterojunction catalysts. The widely employed pseudo-first-order kinetic equation was tested. Figure 5g–i illustrate the reaction kinetics of RhB degradation in the presence of 30 and 50 mg catalysts, as well as 10 and 15 mg/L dye concentrations. From the plots, it can be seen that the photocatalytic degradation of RhB follows the pseudo-first-order kinetics with good correlation constants (R^2^ > 0.95) for all the fitted lines. A good correlation with the pseudo-first-order reaction kinetics for 50 mg 0.5 wt.% Pt@BFO_50_/O-CN_50_ (R^2^ = 0.988) and for 30 mg 0.5 wt.% Pt@BFO_50_/O-CN_50_ (R^2^ = 0.982) was obtained. The calculated pseudo-first-order rate constants for 30 and 50 mg 0.5 wt.% Pt@BFO_50_/O-CN_50_ are 2.38 × 10^−2^ min^−1^ and 4.63 × 10^−2^ min^−1^, respectively. The results further confirmed that the Pt@BFO_50_/O-CN_50_ heterojunction photocatalyst exhibits good photocatalytic activity, which substantiates the corresponding photodegradation efficiency.

#### 2.5.2. Free-Radical Species Assessment

**Figure 6:**
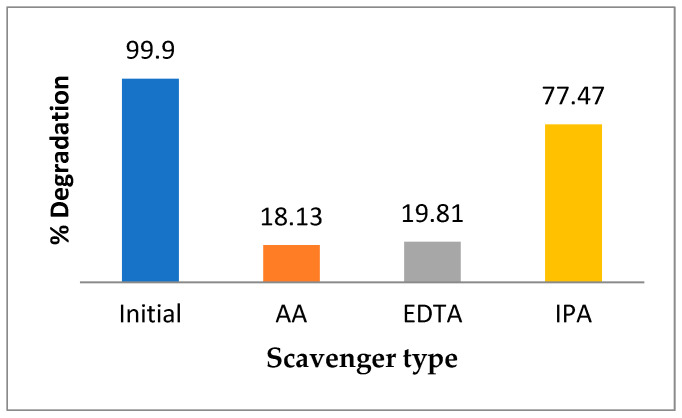
Effect of various trapping agents on free-radical species towards photodegradation of RhB.

Active free-radical species such as holes (h^+^) and hydroxyl (^•^OH) and superoxide (^•^O_2_^−^) radicals are the major actors in the photocatalytic degradation of OMPs. In this study, the roles of these species in the RhB photodegradation over 0.5 wt.% Pt@BFO_50_/O-CN_50_ composites were examined by adding 1 mmol/L each of IPA, EDTA, and AA trapping agents in the way previously described for the photodegradation test. Figure 6 demonstrates that the addition of IPA reduced the degradation efficiency of RhB by 22.43%, indicating that the ^·^OH radical does not have much influence on the photodecomposition of RhB. However, the addition of EDTA and AA resulted in a remarkable decline in the efficiency, demonstrating that scavenging agents h^+^ and ^•^O_2_^−^ radicals noticeably affect the photocatalytic activity of the as-constructed catalyst.

#### 2.5.3. Stability Assessment for the Catalyst

**Figure 7:**
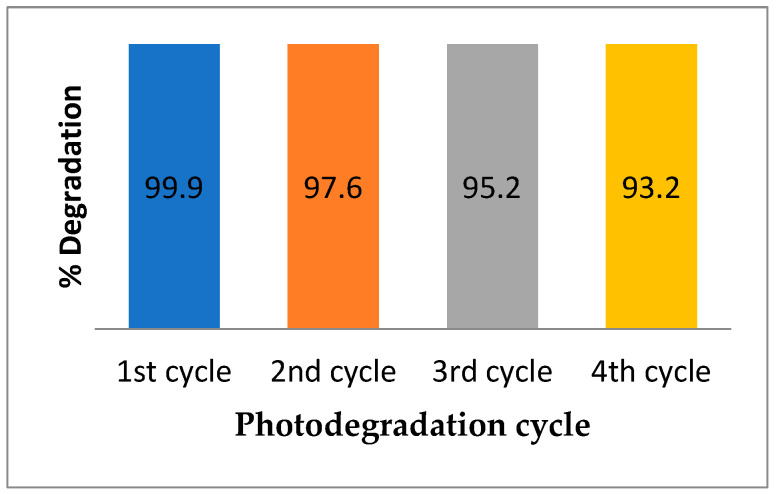
Stability test of the as-constructed catalyst towards photodegradation of RhB.

**Figure 8:**
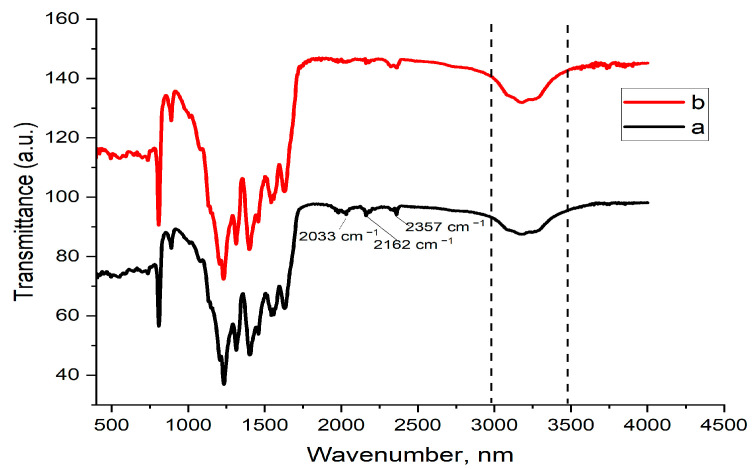
FT-IR spectra of the as-constructed catalyst towards photodegradation of RhB before degradation test (a) and after degradation test cycle 4 (b).

The stability, recyclability, and economic significance of the 0.5 wt.% Pt@BFO_50_/O-CN_50_ heterojunction photocatalyst are considered imperative for the industrial application of the catalyst. Thus, the photocatalyst was recycled four times for RhB photodegradation under visible-light irradiation. Figure 7 shows that the photocatalyst exhibits a photocatalytic efficiency of about 98% after the fourth application, which signifies that 0.5 wt.% Pt@BFO_50_/O-CN_50_ composites preserve their photocatalytic performance without any apparent decline. Additionally, the FT-IR spectra of the as-prepared heterojunction before and after the fourth cycle were compared, and it can be seen from Figure 8 that the catalyst retains its original structure. Hence, the catalyst acquires admirable stability that allows its practical application in water decontamination.

### 2.6. UV-Vis Diffuse Reflectance Spectroscopy (DRS) Analysis

**Figure 9:**
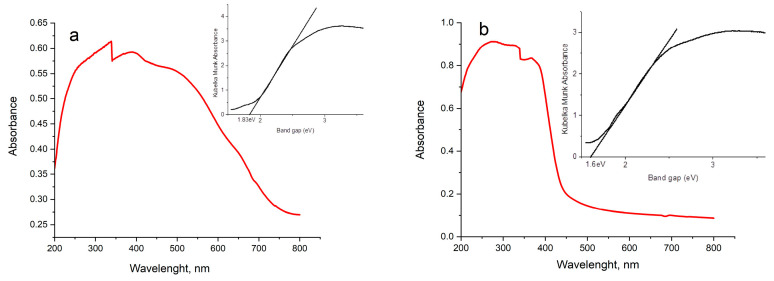

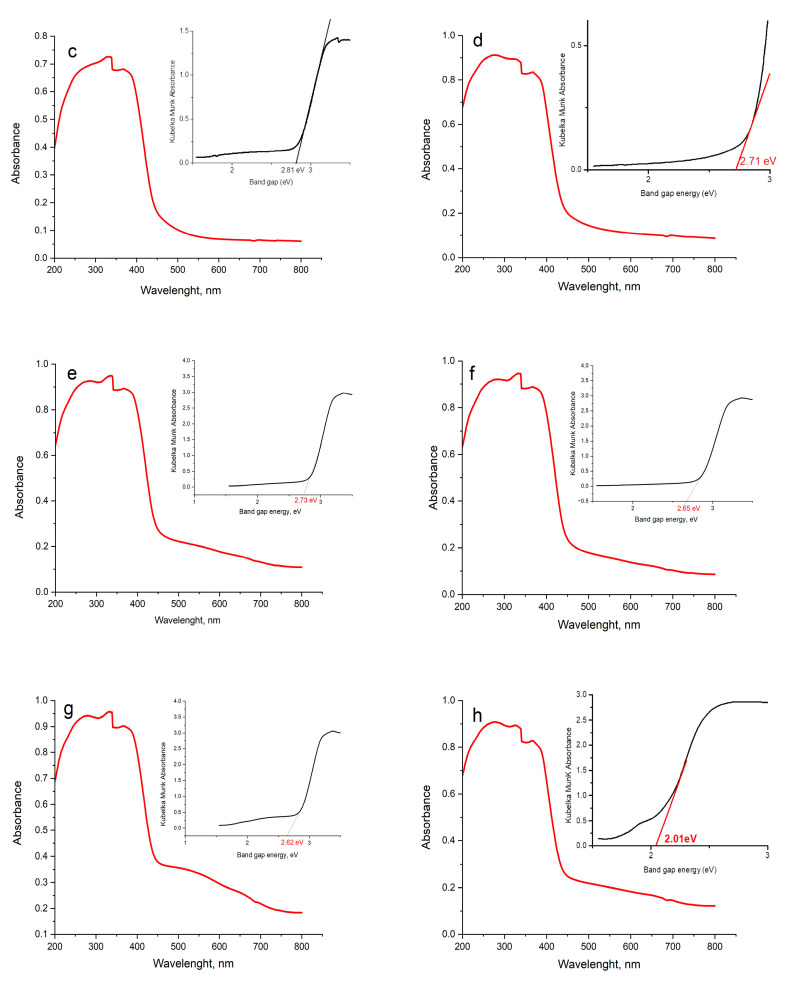
UV-Vis DRS spectra and Tauc plots for (**a**) BFO, (**b**) Pt@BFO, (**c**) O-CN, (**d**) Pt@O-CN, (**e**) BFO_25_/O-CN_75_, (**f**) BFO_50_/O-CN_50_, (**g**) BFO_75_/O-CN_25_, and (**h**) Pt@BFO_50_/O-CN_50_ heterojunction.

The band gaps of the heterojunction system and other as-prepared catalysts were determined by Tauc plot from Kubelka–Munk absorbance obtained from UV-Vis diffuse absorption spectra. The demonstration of broad and strong absorption by all the as-synthesized species within the range of 200-550 nm in Figure 9a–h illustrates the visible-light-responsive capacity of all the catalysts. The UV-Vis DRS spectra of BFO and O-CN in Figure 9a–c registered absorption edges at 677 and 441 nm, respectively. Other edges detected at 775, 457, 454, 468, 473, and 617 nm were credited to Pt@BFO, Pt@O-CN, BFO_25_/O-CN_75_, BFO_50_/O-CN_50_, BFO_75_/O-CN_25_, and Pt@BFO_50_/O-CN_50_ heterojunction, respectively. The computed band gap values are found to be 1.83 eV, 1.60 eV, 2.81 eV, 2.71 eV, 2.73 eV, 2.65 eV, 2.62 eV, and 2.01 eV for BFO, Pt@BFO, O-CN, Pt@O-CN, BFO_25_/O-CN_75_, BFO_50_/O-CN_50_, BFO_75_/O-CN_25_, and Pt@BFO_50_/O-CN_50_ heterojunction, respectively. A similar low band gap value of 1.83 eV for pristine BFO was reported by Gholam et al. [57], and it is obvious that an increase in its mass ratio with O-CN and Pt doping results in a reduction in the band gap. The mesoporous Pt@BFO/O-CN heterojunctions attain an enhanced visible-light absorption in relation to O-CN that enables them to demonstrate improved photocatalytic performance towards the degradation of RhB.

### 2.7. Proposed Photodegradation Mechanism

In order to propose a plausible photodegradation mechanism for the as-constructed Pt@BFO/O-CN heterojunction, VB edge potential (*E_VB_*) and CB edge potential for both BFO and O-CN were estimated using the following equations [58]:

(1) $$E_{VB}={X-E}_{e}+{0.5E}_{g}$$

(2) $$E_{CB}=E_{VB}-E_{g}$$

where *E_VB_* and *E_CB_* are the energies of the valence band and conduction band, *E_e_* is the energy of free electrons vs. hydrogen (4.5 eV), *X* is the absolute electronegativity of catalyst (X_BFO_ = 6.24 eV and X_O-CN_ = 4.73 eV), and *E_g_* is the band gap energy of the catalysts. Applying Equations (1) and (2), *E_VB_* and *E_CB_* for BFO are found to be in the order of +2.655 eV and +0.825 eV, whereas *E_VB_* and *E_CB_* for O-CN are found to be +1.635 eV and −1.17 eV, respectively.

**Figure 10:**
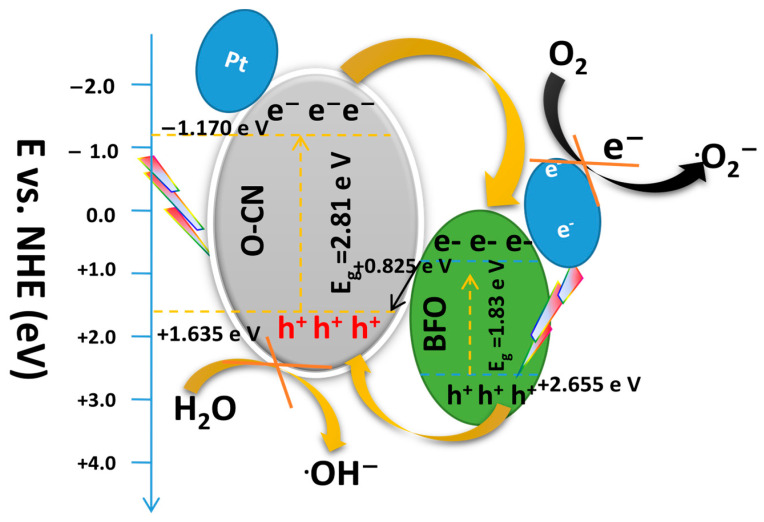
Conventional separation and electron transfer mechanism for Pt@BFO_50_/O-CN_50_ heterojunction photocatalyst.

Using the results for *E_VB_* and *E_CB_* of both BFO and O-CN, and the free-radical trapping test which demonstrated that O^2−^ radical and h^+^ are the most influential species, a conventional charge transfer mechanism for the Pt@BFO/O-CN heterojunction was first considered as schematically illustrated in Figure 10 below.

When light is irradiated on the Pt@BFO/O-CN system, electrons in the BFO and O-CN transition from the VB to the CB leaving behind the corresponding holes in their respective VB [58]. Afterward, e^−^ on the CB of O-CN is spontaneously transferred to the CB of BFO, while the h^+^ on the VB of BFO migrates to the VB of O-CN. However, since the VB potential of O-CN (*E_VB_* = 1.635 eV) is lower than that of ^·^OH/OH^−^ (2.40 eV vs. NHE), the holes on the VB of O-CN cannot oxidize OH^–^ ions to proficiently produce the ^·^OH radicals [59]. Similarly, the photoexcited electrons on the CB of BFO basically cannot produce O_2_^–^ because the CB potential of BFO (*E_CB_* = +0.825 eV) is higher than that of O_2_/O_2_^–^ (−0.33 eV vs. NHE). In addition, this mechanism contradicts the results from the free-radical trapping test which demonstrated that holes and superoxide radicals dominated the photodegradation process. Hence, the above mechanism is wrong, and another possible reaction pathway should be considered.

**Figure 11:**
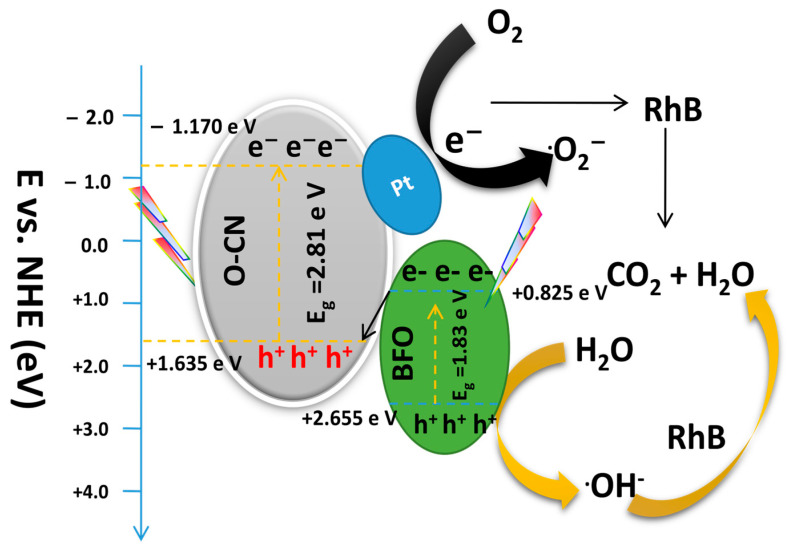
Proposed synergistic separation and electron transfer mechanism for S-scheme Pt@BFO_50_/O-CN_50_ heterojunction photocatalyst.

A possible step-scheme (S-scheme) transfer mechanism is proposed for the degradation of RhB over the Pt@BFO/O-CN heterojunction as schematically illustrated in Figure 11 below. Primarily, when the heterojunction photocatalyst is exposed to visible light, holes and electrons are generated in the corresponding VB and CB of BFO and O-CN. In this charge transfer mechanism, the photoexcited electrons on the CB of O-CN are transferred to the platinum species [60], while the photogenerated electrons on the CB of BFO are recombined with the holes on the VB of O-CN, leaving behind only holes in the VB of BFO, accomplishing efficient charge separation. The holes on the VB of BFO could then be used for the oxidation of RhB since its oxidation potential (*E_VB_* = +2.655 eV) is higher than that of OH/OH^−^ (+2.40 eV vs. NHE), while the transmitted electrons on the surface of Pt would reduce O_2_ to O_2_^−^ radicals that could also be used for the oxidation of the RhB pollutant. This mechanism is consistent with the free-radical trapping test experiments in the section above. The efficient separation of the photogenerated carriers due to the presence of plasmonic Pt particles enhances the photocatalytic activity of the Pt@BFO/O-CN photocatalyst. The S-scheme electron–hole transfer mechanism of Pt@BFO/O-CN can be summarized in the following equations.

## 3. Conclusions

In summary, Pt@BFO/O-CN heterojunctions were successfully constructed using an ultrasonic–hydrothermal method, as confirmed by N_2_ sorption studies, XRD, FT-IR, and SEM-EDS. The photocatalytic performance of the optimized Pt@BFO/O-CN heterojunction was examined by studying the decomposition of an aqueous RhB solution under visible-light irradiation. The Pt@BFO/O-CN heterojunction displays higher photocatalytic activity than the binary BFO/O-CN catalysts and the pristine BFO and O-CN. Superior activity was achieved for the 0.5 wt.% Pt@BFO/O-CN composites when the mass ratio of BFO:O-CN was 50:50. Furthermore, the as-constructed Pt@BFO/O-CN heterojunctions exhibited excellent stability in a four-cycle photocatalytic experiment, and the free-radical scavenging tests revealed that both ^•^O_2_^−^ and h^+^ take a leading role during the photocatalytic reaction. An S-scheme heterojunction was proposed as the plausible charge transfer mechanism for the degradation of RhB over the Pt@BFO/O-CN photocatalyst. Thus, the S-scheme Pt@BFO/O-CN heterojunction photocatalyst is a good candidate in the treatment of industrial wastewater for the mineralization of organic micropollutants, which pose a grievous threat to the environment.

## 4. Materials and Methods

### 4.1. Materials and Reagents 

The following chemicals were used in the synthesis of our catalysts: Urea (CH_4_N_2_O; 60.06 g/mol, 99.5%) was purchased from Carl Roth (Karlsruhe, Germany). Bismuth (III) nitrate pentahydrate (Bi(NO_3_)_3_.5H_2_O; 485.07 g/mol, ≥98%), iron(III) nitrate nonahydrate (Fe(NO_3_)_3_.9H_2_O; 404 g/mol, ≥98%), and diethylene glycol (C_4_H_10_O_3_; 106.12 g/mol, 99%) were procured from Sigma Aldrich (Darmstadt, Germany). Hexachloroplatinic (IV) acid hexahydrate (H_2_PtCl_6_.6H_2_O; 427.8 g/mol, ~40% Pt) was supplied by Merck (Darmstadt, Germany), while rhodamine B (C_28_H_31_CIN_2_O_3_; 479.02 g/mol, ≥98%) was obtained from ThermoFisher (Kandel, Germany). Isopropyl alcohol (C_3_H_7_OH; 60.10 g/mol, 99.7%), hydrogen peroxide (H_2_O_2_; 34.01 g/mol, 30%), methanol (CH_3_OH; 32.04 g/mol, 99.8%), and ethanol (C_2_H_5_OH; 46.07 g/mol, 96%) were obtained from Chemical Ch-C (Iasi, Romania). All chemicals were used as received without further purification, and all photocatalytic experiments started with an RhB dye stock solution of 100 mg/L, which was subsequently diluted to obtain the desired concentrations.

### 4.2. Synthesis of BiFEO_3_ Xerogels

To synthesize the BFO xerogels, an equimolar ratio of Bi^3+^and Fe^3+^ cations from their respective precursors was prepared using isopropyl alcohol (IPA) as a solvent and diethylene glycol (DEG) as a chelating agent. First, a 50 mL mixture of IPA and DEG was sonicated for 15 min, and 0.008 (3.88 g) of bismuth precursor (Bi (NO_3_)_3_⋅5H_2_O) was mixed in the above solution though stirring for 10 min followed by 20 min sonication. Then, 0.008 (1.93 g) of iron precursor (Fe (NO_3_)_3_⋅9H_2_O) was dispersed in the mixture through sonication for 20 min to obtain a homogeneous sol solution. The resulting sol mixture was then magnetically stirred at 70 °C for 30 min, followed by further overnight heating at 80 °C until the BFO xerogels were formed. The resulting xerogels were washed several times with DI water and ethanol to remove the unreacted Bi^3+^, Fe^3+^, and NO^3−^ species. Finally, the BFO xerogels were calcined at 550 °C for 2 h and stored for characterizations and further experiments.

### 4.3. Synthesis of O-g-C_3_N_4_

The g-C_3_N_4_ (CN) was first prepared by thermal polymerization of urea powder [61]. Twenty grams of urea powder was weighed and taken in a porcelain crucible, covered and wrapped with aluminum foil. The sample was placed in a muffle furnace (model) and heated first at 550 °C for 1 h with a ramp rate of 10°/min from room temperature and then maintained at 550 °C for 3 h. The resultant yellow-colored agglomerates were ground into fine particles to obtain the CN powder. Synthesis of O-g-C_3_N_4_ (O-CN) was inspired by Asadzadeh-Khaneghahet al. [62]. Typically, 1 g of the as-prepared CN powder was dispersed in 100 mL 3M H_2_O_2_ (30 wt.%) and magnetically stirred at 150 °C for 30 min. The resulting mixture was cooled down by maintaining it at ambient conditions, and it was collected by washing it several times with water and ethanol, followed by overnight drying at 70 °C. The yellow-orange powdered O-CN was collected and stored for characterizations and further experiments.

### 4.4. Synthesis of BiFeO_3_/O-g-C_3_N_4_

Different mass ratios (BiFeO_3_:O-g-C_3_N_4_; 1:3, 1:1, and 3:1) of the binary BiFeO_3_/O-g-C_3_N_4_ catalysts were previously obtained by simple mechanical crushing of the as-synthesized O-g-C_3_N_4_ and BiFeO_3_ powders inspired by Hu et al. [63]. In a typical procedure, appropriate masses of the BFO and O-CN were mixed and mechanically crushed to obtain the required ratio, followed by calcination of the as-obtained samples at 300 °C for 1 h in a muffle furnace to acquire the desired BiFeO_3_/O-g-C_3_N_4_ heterojunction structures.

### 4.5. Synthesis of Pt@BiFeO_3_/O-g-C_3_N_4_

The synthesis of the 0.5 wt.% Pt@BiFeO_3_/O-g-C_3_N_4_ heterojunction was carried out using an ultrasonic-assisted hydrothermal procedure. Typically, an appropriate mass of H_2_PtCl_6_.6H_2_O was dissolved in 50 mL DI water and sonicated for 15 min. Then, a certain amount of the as-synthesized BiFeO_3_/O-g-C_3_N_4_ was dispersed in the H_2_PtCl_6_.6H_2_O aqueous solution and was sonicated for another 15 min to obtain a homogeneous mixture. The as-obtained solution was then transferred into a Teflon-lined autoclave for hydrothermal treatment at 100 °C for 4 h. The resulting sample was allowed to cool down naturally, washed several times with water and ethanol, and dried in an oven at 80 °C for 4 h before characterizations and further experiments. The same procedure was followed for the syntheses of Pt@BFO and Pt@O-CN composites.

### 4.6. Sample Characterizations

The structures of the xerogel, BFO/O-CN composites, and (0.5 wt.%) Pt@BFO_50_/O-CN_50_ heterojunction were analyzed by Fourier transform infrared (FT-IR) spectroscopy and powder X-ray diffraction (XRD). FT-IR spectra of the as-synthesized samples were detected using a Nicolet Shimadzu IRTracer-100 FT-IR spectrophotometer (Kyoto, Japan) in the 400–4000 cm^−1^ scanning range. XRD patterns were obtained using a Bruker D8 Advance diffractometer (Karlsruhe, Germany; θ-θ type). The parameters for the diffractometer were Cu-Kα radiation (λ = 1.5418 nm), 40 kV, and 40 mA. All XRD patterns were scanned in the 10° to 80° 2θ measurement range at a scanning speed of 5°/min. For the phase identification, Diffracplus Basic software and the PDF-ICDD 2-2008 database were employed, while Diffracplus TOPAS 4.1 software (Karlsruhe, Germany) was used for quantitative analysis.

The textural properties of the as-synthesized samples were analyzed using N2 adsorption–desorption isotherms under 77 K using a Quantachrome Nova 2200e (BET surface area, pore volume, and pore size distribution analyzer; Quantachrome Instruments, Boynton Beach, Florida, USA). The samples were vacuum-degassed at 250 °C for 4 h prior to the analysis. The properties were calculated using the NovaWin software (Boca Raton, FL, USA).

The microstructural morphologies of the as-prepared catalyst samples were examined using a scanning electron microscope (SEM, Scios 2 HIVAC Dual-Beam ultra-high-resolution FIB-SEM; ThermoFisher, Brno, Czech Republic).

A UV–Vis spectrophotometer (Jasco UV-Vis V-550, Oklahoma City, Oklahoma, USA) was employed to record diffuse reflectance spectra of all the as-synthesized samples within the wavelength range of 200 to 800 nm with an integrating sphere assembly.

### 4.7. Photocatalytic Studies

The photocatalytic activity of the as-constructed samples was evaluated at room temperature by RhB degradation in aqueous media under visible light using a Toption photochemical reactor (TOPTION INSTRUMENT Co., Ltd., Xi’an China) equipped with a long arc xenon lamp light source having an emission wavelength of λ > 400 nm. In a typical experiment, 50 mL of RhB solution was mixed with the appropriate mass of the catalyst samples. The mixture was first continuously and uniformly stirred for 60 min in the dark to establish an adsorption–desorption equilibrium between the catalysts and the aqueous RhB solution. Thereafter, the photodegradation proceeded in the presence of the light. To optimize the photocatalysis of the as-constructed heterojunction, operational parameters such as catalyst dose (30 and 50 mg), initial concentration of dye (10 and 15 mg/L), and photodegradation time (0–50 min at 10 min intervals for 10 mg/L; 0–60 min at 15 min intervals for 15 mg/L) were investigated at the pH of 6.65. After irradiation, aliquots were taken at different time intervals and centrifuged in darkness at 2000 rpm for 3 min to separate the catalyst from the solution. The absorbance of each catalyst sample during the photocatalytic experiment was measured at the maximum absorption peak of RhB using a Shimadzu 3600iPlus UV–Vis spectrophotometer (Columbia, Maryland, USA). Similar experiments were carried out on other control samples for comparison. The photodegradation efficiency of RhB (D,%) was calculated by applying Equation (3), while the rate of degradation of the dye pollutant was assumed to obey pseudo-first-order kinetics, and the rate constant for degradation, k, was obtained from the first-order plot Equation (4).

(3) $$Photodegradation\;efficiency\left(D,\%\right)=\frac{A_{0}-A}{A_{0}}\times 100$$

(4) ln⁡(A0A)=kt

where k, A_0_, and A are the pseudo-first-order rate constant, the absorbance at the initial concentration of aqueous RhB solution, and the absorbance at the concentration of RhB at any specific time of visible-light irradiation, respectively.

## Figures and Tables

**Table 1:** Physicochemical properties of the as-prepared samples.

Catalyst	SA_BET_ (m^2^/g)	V_T_ Total Pore Volume (cc/g)	Average Pore Diameter (nm)
O-CN	59.915	0.158	3.732
BFO_25_/O-CN_75_	44.925	0.073	3.941
BFO_50_/O-CN_50_	34.099	0.068	4.464
BFO_75_/O-CN_25_	31.237	0.062	6.465
Pt@BFO_50_/O-CN_50_	28.094	0.054	4.258
BFO	3.584	0.035	10.459

## References

[1] Horppila J. (2019). Sediment nutrients, ecological status and restoration of lakes. Water Res..

[2] Shi K., Song N., Zou Y., Zhu S., Tan H., Tian Y., Zhang B., Yao H., Guan S. (2019). Porphyrin-based porous polyimides: Synthesis, porous structure, carbon dioxide adsorption. Polym. J..

[3] Fan M., Lin Y., Huo H., Liu Y., Zhao L., Wang E., Wei G. (2016). Microbial communities in riparian soils of a settling pond for mine drainage treatment. Water Res..

[4] Yu Y., Duan C., Li S., Peng C., Yang J., Yan K., Bi X., Zou P. (2022). Relationship between environmental pollution and economic development in late-developing regions shows an inverted V. Sci. Total Environ..

[5] Petrie B., Barden R., Kasprzyk-Hordern B. (2015). A review on emerging contaminants in wastewaters and the environment: Current knowledge, understudied areas and recommendations for future monitoring. Water Res..

[6] Murtaza Z.M., Alqassem H.T., Sabouni R., Ghommem M. (2023). Degradation of micropollutants by metal organic framework composite-based catalysts: A review. Environ. Technol..

[7] Tarafdar A., Sirohi R., Balakumaran P.A., Reshmy R., Madhavan A., Sindhu R., Binod P., Kumar Y., Kumar D., Sim S.J. (2022). The hazardous threat of Bisphenol A: Toxicity, detection and remediation. J. Hazard. Mater..

[8] Rodrigues M., Roman M., Heijne A., Sleutels T., Cornelissen E.R., Verliefde A., Cees J.N., Kuntke P. (2023). Characterization of the organic micropollutants behavior during electrochemical ammonia recovery. J. Environ. Chem. Eng..

[9] Schäfer A.I., Akanyeti I., Semião A.J.C. (2011). Micropollutant sorption to membrane polymers: A review of mechanisms for estrogens. Adv. Colloid Interface Sci..

[10] Rostkowski P., Haglund P., Aalizadeh R., Alygizakis N., Thomaidis N., Arandes J.B., Nizzetto P.B., Booij P., Budzinski H., Brunswick P. (2019). The strength in numbers: Comprehensive characterization of house dust using complementary mass spectrometric techniques. Anal. Bioanal. Chem..

[11] Xue P., Zhao Y., Zhao D., Chi M., Yin Y., Xuan Y., Wang X. (2021). Mutagenicity, health risk, and disease burden of exposure to organic micropollutants in water from a drinking water treatment plant in the Yangtze River Delta, China. Ecotoxicol. Environ. Saf..

[12] Han D., Currell M.J. (2017). Persistent organic pollutants in China’s surface water systems. Sci. Total Environ..

[13] Gogoi A., Mazumder P., Tyagi V.K., Tushara Chaminda G.G., An A.K., Kumar M. (2018). Occurrence and fate of emerging contaminants in water environment: A review. Groundw. Sustain. Dev..

[14] Ali E.A., Ismail M.N., Elsabee M.Z. (2020). Chitosan based polyelectrolyte complexes development for anionic and cationic dyes adsorption. Egypt. J. Chem..

[15] Imam S.S., Babamale H.F. (2020). A short review on the removal of rhodamine B dye using agricultural waste-based adsorbents. Asian J. Chem. Sci..

[16] Liu H., Ren X., Chen L. (2016). Synthesis and characterization of magnetic metal—Organic framework for the adsorptive removal of Rhodamine B from aqueous solution. J. Ind. Eng. Chem..

[17] Rodriguez-Narvaez O.M., Peralta-Hernandez J.M., Goonetilleke A., Bandala E.R. (2017). Treatment technologies for emerging contaminants in water: A review. Chem. Eng. J..

[18] Ihaddaden S., Aberkane D., Boukerroui A., Robert D. (2022). Removal of methylene blue (basic dye) by coagulation-flocculation with biomaterials (bentonite and *Opuntia ficus indica*). J. Water Process. Eng..

[19] Abdel-Aziz M.H., Bassyouni M.I., Zoromba M.S., Alshehri A.A. (2019). Removal of Dyes from Waste Solutions by Anodic Oxidation on an Array of Horizontal Graphite Rods Anodes. Ind. Eng. Chem. Res..

[20] Matei D., Katsina A.U., Mihai S., Cursaru D.L., Şomoghi R., Nistor C.L. (2023). Synthesis of Ruthenium-Promoted ZnO/SBA-15 Composites for Enhanced Photocatalytic Degradation of Methylene Blue Dye. Polymers.

[21] Senthil K.P., Varsha M., Senthil R.B., Rangasamy G. (2023). Electrodeionization: Principle, techniques and factors influencing its performance. Environ. Res..

[22] Petrov O., Iwaszczuk N., Kharebava T., Bejanidze I., Pohrebennyk V., Nakashidze N., Petrov A. (2021). Neutralization of Industrial Water by Electrodialysis. Membr. J..

[23] Micheal K., Ayeshamariam A., Devanesan S., Bhuvaneswari K., Pazhanivel T., AlSalhi M.S., Aljaafreh M.J. (2019). Environmental friendly Synthesis of Carbon Nanoplates Supported ZnO Nanorods for enhanced degradation of dyes and organic pollutants with Visible Light Driven Photocatalytic Performance. J. King Saud Univ. Sci..

[24] Wu J., Wang J., Xiao D., Zhu J. (2012). A Method to Improve Electrical Properties of BiFeO_3_ Thin Films. ACS Appl. Mater. Interfaces.

[25] Zhang K., Zhou M., Yang K., Yu C., Mu P., Yu Z., Lu K., Huang W., Dai W. (2022). Photocatalytic H_2_O_2_ production and removal of Cr (VI) via a novel Lu_3_NbO_7_: Yb, Ho/CQDs/AgInS_2_/In_2_S_3_ heterostructure with broad spectral response. J. Hazard. Mater..

[26] Lan Y., Wang Y., Guan Y., Du L., Lv Y. (2023). Synthesis and photocatalytic activity of g-C_3_N_4_/BiVO_4_/CNTs composites. Mater. Lett..

[27] Shi H., He R., Sun L., Cao G., Yuan X., Xia D. (2019). Band gap tuning of g-C_3_N_4_ via decoration with AgCl to expedite the photocatalytic degradation and mineralization of oxalic acid. J. Environ. Sci..

[28] Chen J., Yang Q., Zhong J., Li J., Hu C., Deng Z., Duan R. (2018). In-situ construction of direct Z-scheme Bi_2_WO_6_/g-C_3_N_4_ composites with remarkably promoted solar-driven photocatalytic activity. Mater. Chem. Phys..

[29] Ramadan W., Feldhoff A., Bahnemann D. (2021). Assessing the photocatalytic oxygen evolution reaction of BiFeO_3_ loaded with IrO_2_ nanoparticles as co catalyst. Sol. Energy Mater. Sol. Cells.

[30] Soltani T., Entezari M.H. (2013). Solar photocatalytic degradation of RB_5_ by ferrite bismuth nanoparticles synthesized via ultrasound. Ultrason. Sonoc..

[31] Maleki H. (2018). Photocatalytic activity, optical and ferroelectric properties of Bi 0.8 Nd 0.2 FeO_3_ nanoparticles synthesized by sol-gel and hydrothermal methods. J. Magn. Magn. Mater..

[32] Wani W.A., Kundu S., Ramaswamy K. (2020). Optimizing phase formation of BiFeO_3_ and Mn-doped BiFeO_3_ nanoceramics via thermal treatment using citrate precursor method. SN Appl. Sci..

[33] Dhanalakshmi R., Muneeswaran M., Shalini K., Giridharan N.V. (2016). Enhanced photocatalytic activity of La-substituted BiFeO_3_ nanostructures on the degradation of phenol red. Mater Lett..

[34] Ong W.J., Tan L.L., Ng Y.H., Yong S.T., Chai S.P. (2016). Graphitic carbon nitride (gC_3_N_4_)-based photocatalysts for artificial photosynthesis and environmental remediation: Are we a step closer to achieving sustainability?. Chem. Rev..

[35] Mousavi M., Habibi-Yangjeh A., Rahim P.S. (2018). Review on magnetically separable graphitic carbon nitride-based nanocomposites as promising visible-lightdriven photocatalysts. J. Mater. Sci. Mater. Electron..

[36] Wang Y., Sun J., Yao Y., Li Z., Meng X. (2022). Investigation of Photo(electro)catalytic water splitting to evolve H_2_ on Pt-g-C_3_N_4_ nanosheets. Int. J. Hydrog Energy.

[37] Liu S., Li D., Sun H., Ang H.M., Tadé M.O., Wang S. (2016). Oxygen functional groups in graphitic carbon nitride for enhanced photocatalysis. J. Colloid Interface Sci..

[38] Ilkaeva M., Krivtsov I., García J.R., Díaz E., Ordóñez S., García-López E.I., Malato S. (2018). Selective photocatalytic oxidation of 5-hydroxymethyl-2-furfural in aqueous suspension of polymeric carbon nitride and its adduct with H_2_O_2_ in a solar pilot plant. Catal. Today.

[39] Besteiro L.V., Yu P., Wang Z., Holleitner A.W., Hartland G.V., Wiederrecht G.P., Govorov A.O. (2019). The fast and the furious: Uitrafast hot electrons in pasmonic metastructures. Size Struct. Matter Nanotoday.

[40] Usman A.K., Cursaru D.-L., Brănoiu G., Şomoghi R., Manta A.-M., Matei D., Mihai S. (2022). A Modified Sol–Gel Synthesis of Anatase {001}-TiO_2_/Au Hybrid Nanocomposites for Enhanced Photodegradation of Organic Contaminants. Gels.

[41] Li C., Zhu D., Cheng S., Zuo Y., Wang Y., Ma C., Dong H. (2022). Recent research progress of bimetallic phosphides-based nanomaterials as cocatalyst for photocatalytic hydrogen evolution. Chin. Chem. Lett..

[42] Yu Z., Yang K., Yu C., Lu K., Huang W., Xu L., Zou L., Wang S., Chen Z., Hu J. (2022). Steering unit cell dipole and internal electric field by highly dispersed Er atoms embedded into NiO for efficient CO_2_ photoreduction. Adv. Funct. Mater..

[43] Liu X., Huang W.Y., Zhou Q., Chen X.R., Yang K., Li D., Dionysiou D.D. (2021). Ag-decorated 3D flower-like Bi_2_MoO_6_/rGO with boosted photocatalytic performance for removal of organic pollutants. Rare Met..

[44] Quiroz J., Barbosa E.C.M., Araujo T.P., Fiorio J.L., Wang Y.C., Zou Y.C., Mou T., Alves T.V., Oliveira C.D., Wang B. (2018). Camargo Controlling reaction selectivity over hybrid plasmonic nanocatalysts. Nano. Lett..

[45] Wang P.-F., Chen K., Ma S., Wang W., Qiu Y.-H., Ding S.-J., Liang S., Wang Q.-Q. (2020). Asymmetric synthesis of Au-CdSe core-semishell nanorods for plasmon-enhanced visible-light driven hydrogen evolution. Nanoscale.

[46] Zhang N., Qi M., Yuan L., Fu X., Tang Z., Gong J., Xu Y. (2019). Broadband light harvesting and unidirectional electron flow for efficient electron accumulation for hydrogen generation. Angew. Chem. Int. Ed..

[47] Wu S., Zhang J., Liu X., Lv S., Gao R., Cai W., Fu C. (2019). Micro-Area Ferroelectric, Piezoelectric and Conductive Properties of Single BiFeO_3_ Nanowire by Scanning Probe Microscopy. Nanomater.

[48] Wang J. (2003). Epitaxial BiFeO_3_ Multiferroic Thin Film Heterostructures. Science.

[49] Patnaik S., Sahoo D.P., Parida K. (2018). An overview on Ag modified g-C3N4 based nanostructured materials for energy and environmental applications. Renew. Sust. Energ. Rev..

[50] Jaramillo C.A., Navío J.A., Hidalgo M.C., Macias M. (2018). ZnO and Pt-ZnO photocatalysts: Characterization and photocatalytic activity assessing by means of three substrates. Catal. Today.

[51] Rodríguez-Padrón D., Puente-Santiago A.R., Balu A.M., Muñoz-Batista M.J., Luque R. (2019). Continuous Flow Synthesis of High Valuable N-Heterocycles via Catalytic Conversion of Levulinic. Front. Chem..

[52] Liu H., Cheng D.-G., Chen F., Zhan X. (2020). 2D porous N-deficient g-C3N4 nanosheet decorated with CdS nanoparticles for enhanced visible-light-driven photocatalysis. ACS Sustain. Chem. Eng..

[53] Guo F., Li M., Ren H., Huang X., Shu K., Shi W., Lu C. (2019). Facile bottom-up preparation of Cl-doped porous g-C3N4 nanosheets for enhanced photocatalytic degradation of tetracycline under visible light. Sep. Purif. Technol..

[54] Shao B., Liu X., Liu Z., Zeng G., Liang Q., Liang C., Cheng Y., Zhang W., Liu Y., Gong S. (2019). A novel double Z-scheme photocatalyst Ag_3_PO_4_/Bi_2_S_3_/Bi_2_O_3_ with enhanced visible-light photocatalytic performance for antibiotic degradation. Chem. Eng. J..

[55] Shekofteh-Gohari M., Habibi-Yangjeh A., Abitorabi M., Rouhi A. (2018). Magnetically separable nanocomposites based on ZnO and their applications in photocatalytic processes: A review. Crit. Rev. Environ. Sci. Technol..

[56] Yang L., Wang P., Yin J., Wang C., Dong G., Wang Y., Ho W. (2019). Engineering of Incorporation the Reduced Graphene Oxide on Nanosheet–g-C_3_N_4_/Perylene Imide Heterojunction for Enhanced Photocatalytic Redox Performance. Appl. Catal. B Environ..

[57] Gholam T., Zheng L.R., Wang J.O., Qian H.J., Wu R., Wang H.-Q. (2019). Synchrotron X-ray Absorption Spectroscopy Study of Local Structure in Al-Doped BiFeO_3_ Powders. Nanoscale Res. Lett..

[58] Tong R., Wang X., Zhou X., Liu Q., Wang H., Peng X., Lund P.D. (2017). Cobalt-Phosphate modified TiO_2_/BiVO_4_ nanoarrays photoanode for efficient water splitting. Int. J. Hydrog Energy.

[59] Ahmad M., Ali R., Rehman A., Ali A., Sultana I., Ali I., Asif M. (2020). Insight into the Structural, Electrical, and Magnetic Properties of Al-Substituted BiFeO_3_ Synthesised by the Sol–Gel Method. Z. Für Nat. A.

[60] Carneiro J.O., Samantilleke A.P., Parpot P., Fernandes F., Pastor M., Correia A., Luís A., Chivanga B.A., Teixeira A.V. (2016). Visible Light Induced Enhanced Photocatalytic Degradation of Industrial Effluents (Rhodamine B) in Aqueous Media Using TiO_2_ Nanoparticles. J. Nanomater..

[61] Thommes M., Kaneko K., Neimark A.V., Olivier J.P., Rodriguez-Reinoso F., Rouquerol J., Sing K.S.W. (2015). Physisorption of gases, with special reference to the evaluation of surface area and pore size distribution (IUPAC Technical Report). Pure Appl. Chem..

[62] Anh H.Q., Le T.P.Q., Da Le N., Lu X.X., Duong T.T., Garnier J., Rochelle-Newall E., Zhang S., Oh N.-H., Oeurng C. (2021). Antibiotics in surface water of East and Southeast Asian countries: A focused review on contamination status, pollution sources, potential risks, and future perspectives. Sci. Total Environ..

[63] Vatanpour V., Paziresh S., Dehqan A., Asadzadeh-Khaneghah S., Habibi-Yangjeh A. (2021). Hydrogen peroxide treated g-C_3_N_4_ as an effective hydrophilic nanosheet for modification of polyethersulfone membranes with enhanced permeability and antifouling characteristics. Chemosphere.

